# Association between prealbumin levels and the risk of osteoporosis among individuals aged 50 and above in Southwest China: a retrospective case–control study

**DOI:** 10.3389/fmed.2025.1695044

**Published:** 2025-12-17

**Authors:** Xu Zhao, Chenhui Cai, Chao Tang, Zaoqing Zhang, Xianming Huang, Tongwei Chu

**Affiliations:** 1 Department of Orthopedics, Xinqiao Hospital, Third Military Medical University (Army Medical University), Chongqing, China; 2 Unit 66397 of the People’s Liberation Army, Beijing, China; ^3^Department of Orthopedics, The Affiliated Hospital of Southwest Medical University, Luzhou, China

**Keywords:** osteoporosis, bone mineral density, risk factor, prealbumin, hemoglobin A1c, case–control

## Abstract

**Background:**

Suboptimal nutritional status has been significantly correlated with the risk of osteoporosis in postmenopausal women. Low prealbumin levels are considered independent factors associated with decreased bone mineral density (BMD). However, most studies were conducted exclusively on postmenopausal women. Therefore, further investigations are warranted to determine the extent to which these findings can be extrapolated to the male and general population.

**Objective:**

To investigate the association between prealbumin levels and BMD among individuals aged 50 and above residing in southwestern China.

**Methods:**

A case–control study was conducted. A total of 534 individuals aged ≥ 50 years who underwent dual-energy X-ray absorptiometry scanning between 2018 and 2023 at the Second Affiliated Hospital of the Army Medical University were included in this study. Prealbumin levels; hemoglobin A1c levels; alkaline phosphatase, aspartate aminotransferase, and alanine aminotransferase levels; age; sex; body mass index; and lifestyle factors were recorded by reviewing medical records. We defined osteoporosis in individuals aged ≥ 50 years according to the World Health Organization criteria, which recommends a T score ≤ − 2.5. Multivariable logistic regression was used to analyze the risk factors for osteoporosis, particularly the association between prealbumin levels and osteoporosis.

**Results:**

A total of 534 participants who met the inclusion criteria were recruited. Univariate analysis revealed statistically significant differences in age, body mass index, sex, type of current residence, physical activity, prealbumin, albumin, hemoglobin levels, and Hemoglobin A1c levels (*p* < 0.05). Bivariate correlation analysis revealed that Hemoglobin A1c (*r* = −0.287, *p* < 0.01) was negatively correlated with BMD in participants, while albumin (*r* = 0.206, *p* < 0.01), prealbumin (*r* = 0.292, *p* < 0.01) and Hemoglobin (*r* = 0.255, *p* < 0.01) were positively correlated with BMD. Multiple logistic regression analysis revealed prealbumin level as a significant determinant of decreased BMD (OR, odds ratio = 2.317; 95% CI, confidence interval: 1.439–3.731; *p* < 0.05).

**Conclusion:**

Our findings demonstrate that low prealbumin levels are significantly associated with an increased risk of osteoporosis among adults aged ≥50 years in southwestern China. Individuals with this risk factor should receive regular BMD monitoring and early targeted interventions to prevent osteoporosis.

## 1 Background

Metabolic bone disorders, such as osteoporosis, are prevalent globally, including the southwestern region of China. It is a diagnosable and treatable subclinical condition characterized by decreased bone mineral density (BMD) and quality, damaged microstructure, and increased fragility resulting from various contributing factors. Delayed intervention and treatment are likely to result in fractures. With the accelerated arrival of population aging, it is obvious that osteoporosis and osteoporotic fractures will constitute a huge medical, public health and economic burden worldwide. As the world’s largest developing country, China has a huge population base and many underdeveloped areas, which makes the management of osteoporosis more challenging. Data indicates that the overall prevalence rate of osteoporosis among all adults in mainland China is approximately 7%. Specifically, the prevalence rates are 22.5% among men aged 50 or above and 40.1% among women in the same age group (1). Despite the gradual establishment of a multi-level prevention and control system through policy guidance, technological innovation, and public health initiatives, significant challenges such as the fragmented primary healthcare system and the limited accessibility of dual-energy X-ray absorptiometry (DXA) scans persist, particularly in remote areas. If more accessible methods, such as analyses of population characteristics and blood biochemical indicators, could be employed to identify high-risk populations for osteoporosis and recommend further targeted osteoporosis screening, this strategy would likely play a critical role in the prevention and early intervention of osteoporosis, thereby highlighting its substantial practical implications. Many studies have found that low BMD is associated with aging, low body weight, lack of exercise, low income, excessive drinking, diabetes, and pernicious anemia (2–4). Moreover, insufficient nutrition is considered a risk factor for osteoporosis, and some studies suggest that prealbumin is more suitable than albumin as a malnutrition biomarker (5).

Prealbumin, also known as transthyretin, is synthesized in hepatic cells. Its main function is to bind and transport thyroid hormones throughout the body (6). Low prealbumin levels often indicate malnutrition, inflammation, and liver-related diseases, owing to its short half-life and limited body pool. This makes it a reliable protein-status marker and an excellent indicator of malnutrition (7). Several studies have suggested that a low prealbumin level may be a risk factor for osteoporosis, although the underlying mechanism remains unclear. As early as 1993, it was proposed that patients with osteoporosis exhibit a significant reduction in prealbumin levels compared with those in healthy women of the same age, along with decreased levels of nutritional biochemical markers, such as retinol-binding protein. These findings suggest a potential association between osteoporosis and nutritional deficiency (8). A previous cross-sectional study reported a significant association between prealbumin levels and the development of osteoporosis. In adult women, there is a positive association between BMD and low prealbumin levels, which may be triggered by chronic inflammation during osteoporosis (9). Another study revealed a noteworthy correlation between decreased prealbumin levels and increased susceptibility to osteoporosis among individuals diagnosed with type 2 diabetes mellitus (10). A recent cross-sectional study investigating the relationship between gastrointestinal health, serum proteins, and BMD in postmenopausal women also suggested a potential negative correlation between prealbumin levels and BMD (11). It is important to note that all the aforementioned studies were conducted within the Chinese population. These studies have highlighted a potential close association between low prealbumin levels and the development of osteoporosis. However, the previous studies have predominantly examined the relationship between prealbumin levels and the risk of osteoporosis in postmenopausal women. Our aim is to extend the scope of this study to a broader cohort of middle-aged and elderly individuals, including both men and women. This approach not only addresses the research gap concerning the role of prealbumin in male osteoporosis but also provides valuable insights into how prealbumin levels influence osteoporosis risk across diverse populations, rather than being limited to the hormone-driven mechanisms observed in postmenopausal women.

## 2 Methods

### 2.1 Study participants

We included 534 participants residing in the southwestern region of China (with a mean age of 62.93 ± 9.00 years; range: 50–90 years) who had similar lifestyle choices and underwent DXA scanning at the Second Affiliated Hospital Army Military Medical University between 2018 and 2023. Participants with previous medical conditions, such as cancer, rheumatoid arthritis, autoimmune disorders, chronic liver and kidney diseases, anorexia nervosa, cachexia, fractures, coronary heart disease, stroke, or stage four–five chronic kidney disease, were excluded from this study. In addition, those receiving hormone replacement therapy and glucocorticoid drugs were excluded. Ethical approval for this retrospective study was granted by the Medical Ethics Committee of the Second Affiliated Hospital, Army Military Medical University (2023–157-02). The committee waived the requirement for written informed consent because the study involved anonymized analysis of pre-existing medical records without any additional interventions or risks to participants. All patient identifiers were removed prior to data analysis to ensure confidentiality.

### 2.2 Definition of osteoporosis

BMD in the lumbar spine (L1–L4) was assessed using DXA. BMD was measured in grams per square centimeter (g/cm^2^), and T-scores were calculated. According to the criteria established by the World Health Organization, osteoporosis was diagnosed when the BMD T-score reached ≤ − 2.5 (12).

### 2.3 Sociodemographic factors

Data on study participants were obtained by reviewing their past medical records. The age categories were as follows: 50–59, 60–69, and over 70 years. Participants who engaged in physical activity less than three times a week were considered less physically active, whereas those who exercised more than three times a week were considered to have regular exercise habits. The living environment was categorized into urban and rural settings. Anthropometric measurements, including body weight and height, were obtained by well-trained examiners following standardized protocols. Body mass index (BMI) was calculated by dividing the weight (in kilograms) by the square of height (in meters). These factors align with known osteoporosis predictors (13, 14).

### 2.4 Biochemical measurements

After a 12-h overnight fasting period, blood samples were collected from the antecubital veins of the participants. The measured parameters included albumin, prealbumin, hemoglobin A1c, hemoglobin, alkaline phosphatase (ALP), aspartate aminotransferase (AST), and alanine aminotransferase (ALT) levels. These were selected based on their reported associations with bone metabolism and disease progression (4, 9, 15–17). The experiments were conducted at the Laboratory of Analytical Biochemistry, Second Affiliated Hospital of the Army Military Medical University, Chongqing, China.

For the analysis, a prealbumin level of 200 mg/L was used as the threshold to define low prealbumin status. This cutoff was adopted from the standard normal reference range (200–400 mg/L) established and validated by our institutional clinical laboratory for the local adult population.

### 2.5 Statistical analysis

Statistical analyses were conducted using IBM SPSS Statistics ver. 25.0. Normally distributed data are presented as means with standard deviations, whereas variables with a skewed distribution are reported as medians (interquartile ranges). Categorical variables are presented as frequencies. Firstly, we conducted a univariate difference analysis on the data using the Mann–Whitney U test and chi-square test to initially screen out the differential variables related to osteoporosis. Among them, the Mann–Whitney U test was used to determine the intergroup differences between the control group and the osteoporosis group in continuous variables. The categorical variables were expressed as percentages (%) and were compared using the chi-square test. Considering that the reference values for ALP and hemoglobin vary by sex and may potentially affect the accuracy of the results, we performed subgroup analyses stratified by sex to assess whether gender affects these biochemical indicators. Subsequently, we employed bivariate correlation analysis to examine the correlations between BMD and prealbumin, albumin, hemoglobin levels, as well as glycated hemoglobin, further revealing the strength and direction of the associations among the variables. Finally, a multivariable regression model was employed to further clarify the independent association between each selected differential variable and the development of osteoporosis, thus enabling the identification of independent risk factors associated with osteoporosis. Furthermore, to evaluate the predictive performance of the significant risk factors identified in the regression analysis, we performed two complementary receiver operating characteristic (ROC) analyses. First, the discriminatory capacity of prealbumin alone was evaluated, with the optimal cutoff value determined by maximizing the Youden’s index (J = sensitivity + specificity − 1). Second, a composite predictive model was developed based on the predicted probabilities derived from the final multivariate logistic regression. The area under the ROC curve (AUC) was used to quantify the performance of the composite model.

### 2.6 Sample size estimation

*A priori* sample size calculation was performed for this case–control study using G*Power software (version 3.1.9.7). The calculation was based on the comparison of prealbumin levels between the osteoporosis and control groups. Based on a previous study (9), which reported an odds ratio (OR) of 2.75 for low prealbumin in women with osteoporosis, with a prevalence of 32.6% in the normal population, we determined the required sample size. With a two-sided alpha (*α*) of 0.05, a statistical power (1-*β*) of 0.90, and a case-to-control allocation ratio of 1:1, the analysis indicated a minimum requirement of 172 participants (86 per group). The final study sample comprised 534 participants.

## 3 Results

### 3.1 Comparison of demographic characteristics between individuals with and without osteoporosis

**Figure 1:**
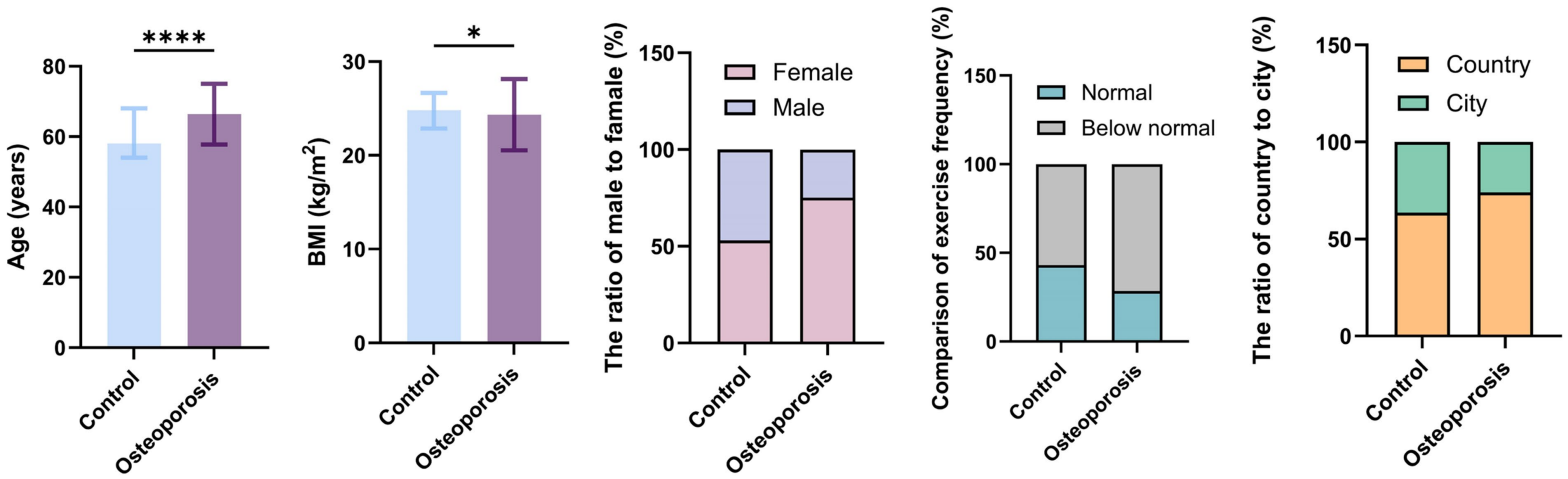
Bar chart comparing general characteristics between control and osteoporosis groups. This bar chart provides a visual comparison of participant characteristics. Data for continuous variables (Age and BMI) are presented as the median with interquartile range for each group. Data for categorical variables (Sex, Type of current residence, and Physical activity) are presented as percentages. ****p < 0.0001, ***p < 0.001, **p < 0.01, *p < 0.05.

The assessment of osteoporosis risk factors was conducted via a stratified analysis of the control (T-Score ≥ − 2.5; *n* = 337) and osteoporosis (T-Score < −2.5; *n* = 197) groups. The baseline characteristics of both groups are presented in Table 1 and Figure 1. The osteoporosis group had a higher mean age but a lower BMI compared to the control group, suggesting that advanced age and low BMI may contribute to the development of osteoporosis. To compare BMD values across different age and BMI categories, a one-way ANOVA was performed across the three age categories (50–59, 60–69, and ≥70 years) and three BMI categories (underweight, normal weight, and obese), followed by *post hoc* tests with Bonferroni correction for multiple comparisons. The findings revealed a positive correlation between aging and incidence of osteoporosis, with a significant increase observed after 60 years of age. Furthermore, compared with individuals with normal weight or overweight status, those classified as underweight were more susceptible to developing osteoporosis. No significant differences were found between individuals with normal weight and those who were overweight. The osteoporosis group had a higher proportion of women, those with a sedentary lifestyle, and rural residence compared with that in the control group.

**Table 1:** General characteristics of the participants with and without osteoporosis.

Characteristics	Control group (*n* = 337)	Osteoporosis group (*n* = 197)	*z*/*x*^2^	*p*
Age (years)	58.00 (54.00, 68.00)	67.00 (59.00, 73.00)	6.956	<0.05
BMI (kg/m2)	24.80 (22.85, 26.67)	24.14 (21.97, 26.34)	−2.351	<0.05
Sex				
Female	179 (53.11%)	148 (75.13%)	25.375	<0.05
Male	158 (46.88%)	49 (24.83%)		
Type of current residence				
Country	214 (63.50%)	146 (74.11%)	6.371	<0.05
City	123 (36.50%)	51 (25.89%)		
Physical activity				
Less than three times a week	192 (56.97%)	141 (71.57%)	11.291	<0.05
Three times a week or more	145 (43.03%)	56 (28.43%)		

### 3.2 Comparison of serum biochemical parameters between individuals with and without osteoporosis

**Figure 2:**
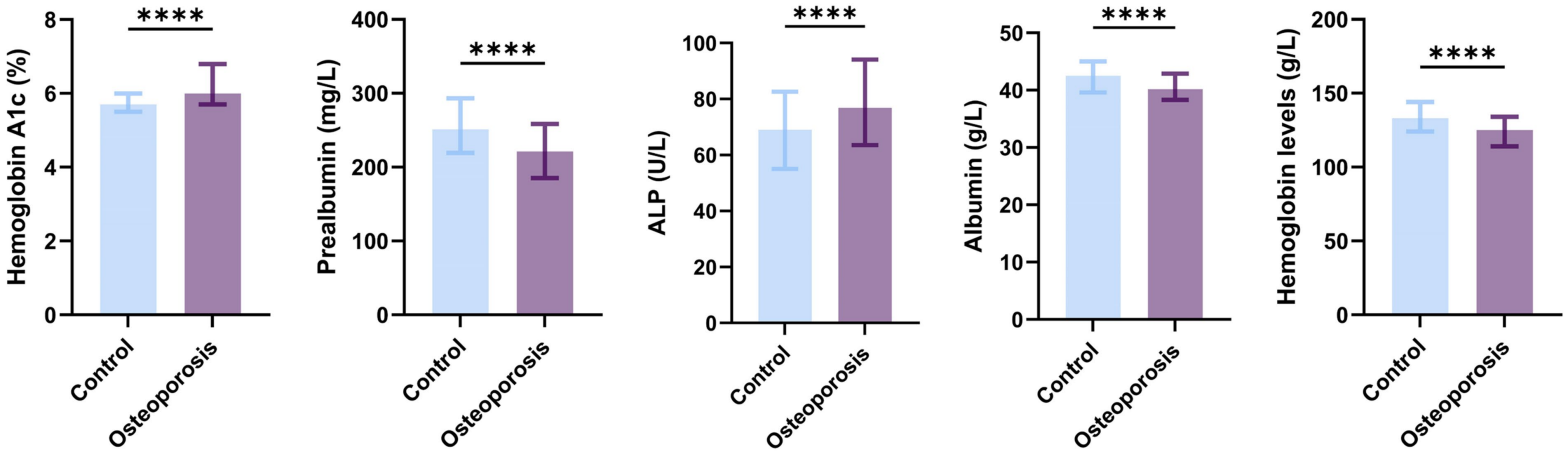
Bar chart comparing biochemical markers between control and osteoporosis groups. This bar chart illustrates a comparative analysis of biochemical profiles between the osteoporosis group and the control group. The measured indicators included hemoglobin A1c (%), prealbumin (mg/L), alkaline phosphatase (U/L), albumin (g/L), and hemoglobin (g/L). All parameters exhibited statistically significant intergroup differences ****p < 0.0001, ***p < 0.001, **p < 0.01, *p < 0.05.

The differences in biochemical indices between the osteoporosis and control groups are presented in Table 2 and Figure 2. Overall, significant associations were observed between the occurrence of osteoporosis and increased levels of hemoglobin A1c and ALP, as well as decreased levels of hemoglobin, albumin, and prealbumin (all *p* < 0.05).

**Table 2:** Biochemical markers of the participants with and without osteoporosis.

Characteristics	Control group (*n* = 337)	Osteoporosis group (*n* = 197)	*z*	*p*
Hemoglobin A1c levels (%)	5.70 (5.50, 6.00)	6.00 (5.70, 6.80)	6.200	<0.05
Prealbumin (mg/L)	251.00 (219.00, 293.00)	221.00 (185.00, 258.50)	−6.202	<0.05
ALP (U/L)	68.70 (55.00, 81.85)	77.70 (63.80, 95.90)	5.508	<0.05
Albumin (g/L)	42.50 (39.60, 45.00)	40.20 (38.30, 42.90)	−5.273	<0.05
Hemoglobin levels (g/L)	133.00 (124.00, 144.00)	125.00 (114.00, 134.00)	−6.056	<0.05

**Figure 3:**
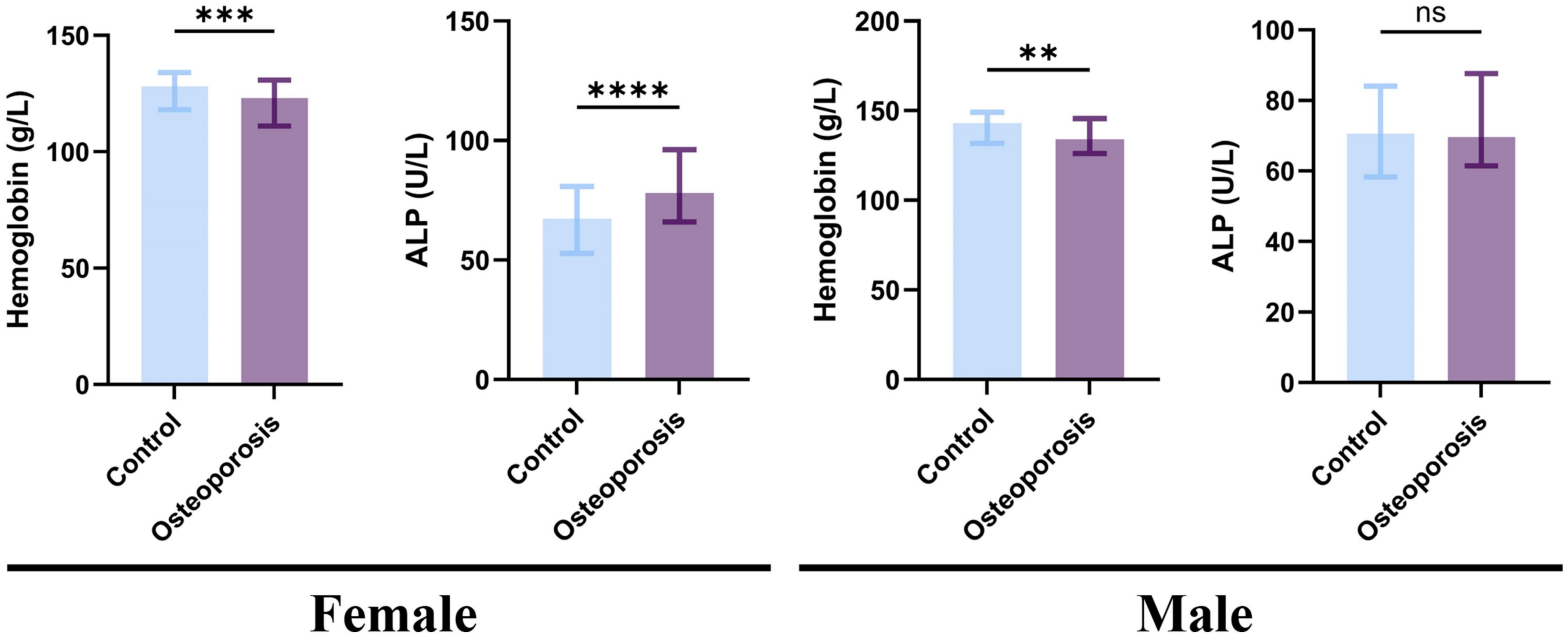
Bar chart comparing hemoglobin and ALP levels between control and osteoporosis groups, stratified by sex. Distributions of hemoglobin and ALP in the study groups by sex. Statistically significant differences were observed for hemoglobin levels in both men and women, and for ALP levels in women. The difference in ALP for men was not significant. ****p < 0.0001, ***p < 0.001, **p < 0.01, *p < 0.05.

Considering the variations in reference values for ALP and hemoglobin across sexes may affect the accuracy of the findings, subgroup analyses stratified by sex were performed. The significant differences between the sexes are presented in Table 3 and Figure 3. ALP is widely recognized as a crucial indicator of accelerated bone turnover in postmenopausal women (18). ALP levels were significantly higher in women with osteoporosis than in controls [control group: 67.30 (52.80, 80.80) U/L; osteoporosis group: 78.00 (65.98, 96.18) U/L; z = 4.833, *p* < 0.05], whereas no significant difference was observed among men. Furthermore, the research results show that, regardless of gender, hemoglobin levels are significantly associated with the occurrence of osteoporosis.

**Table 3:** Comparison of hemoglobin and ALP by sex and group.

Male				
Characteristics	Control group (*n* = 158)	Osteoporosis group (*n* = 49)	*z*	*p*
Hemoglobin (g/L)	143.00 (131.75, 149.00)	134.00 (126.00, 145.50)	−2.617	<0.05
ALP (U/L)	70.55 (58.30, 84.03)	61.45 (69.60, 87.65)	1.065	0.287

Female				
Characteristics	Control group (*n* = 179)	Osteoporosis group (*n* = 148)	*z*	–
Hemoglobin (g/L)	128.00 (118.00, 134.00)	123.00 (111.00, 130.00)	−3.345	<0.05
ALP (U/L)	67.30 (52.80, 80.80)	78.00 (65.98, 96.18)	4.833	<0.05

### 3.3 Correlation analysis between BMD and hemoglobin A1c, albumin, Prealbumin, and hemoglobin

**Figure 4:**
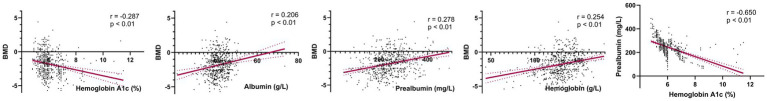
Correlation analysis of different biochemical indicators with BMD and prealbumin. This series of scatter plots illustrates the correlations of BMD with hemoglobin A1c, albumin, prealbumin, and hemoglobin, respectively, along with the correlation between prealbumin and hemoglobin A1c. BMD showed positive correlations with hemoglobin, albumin, and prealbumin, but a negative correlation with hemoglobin A1c. A negative correlation was also observed between prealbumin and hemoglobin A1c.

To comprehensively investigate the risk factors associated with osteoporosis, we performed a bivariate correlation analysis. Bivariate correlation analysis was used to compare the correlation between different variables. Table 4 and Figure 4 show a significant positive correlation between prealbumin, albumin, and hemoglobin levels and BMD. Notably, an inverse relationship was observed between BMD and hemoglobin A1c level (*p* < 0.05). Simultaneously, the results revealed that the Pearson’s correlation index of prealbumin and hemoglobin A1c was −0.650, which indicated highly negative correlation.

**Table 4:** Bivariate correlation analysis between prealbumin and albumin, hemoglobin, and glycosylated hemoglobin.

Characteristics	BMD	Hemoglobin A1c	Albumin	Prealbumin	Hemoglobin
BMD	1				
Hemoglobin A1c	−0.287^**^	1			
Albumin	0.206^**^	−0.346^**^	1		
Prealbumin	0.292^**^	−0.650^**^	0.490^**^	1	
Hemoglobin	0.254^**^	−0.302^**^	0.444^**^	0.469^**^	1

Bivariate correlation analysis was used to compare the correlation between different variables. Pearson correlation index of prealbumin and Hba1c was -0.650, which was highly negative correlation. ^**^ Indicates *p* < 0.01. Abbreviations: BMD, bone mineral density.

### 3.4 Analysis of risk factors for osteoporosis

**Figure 5:**
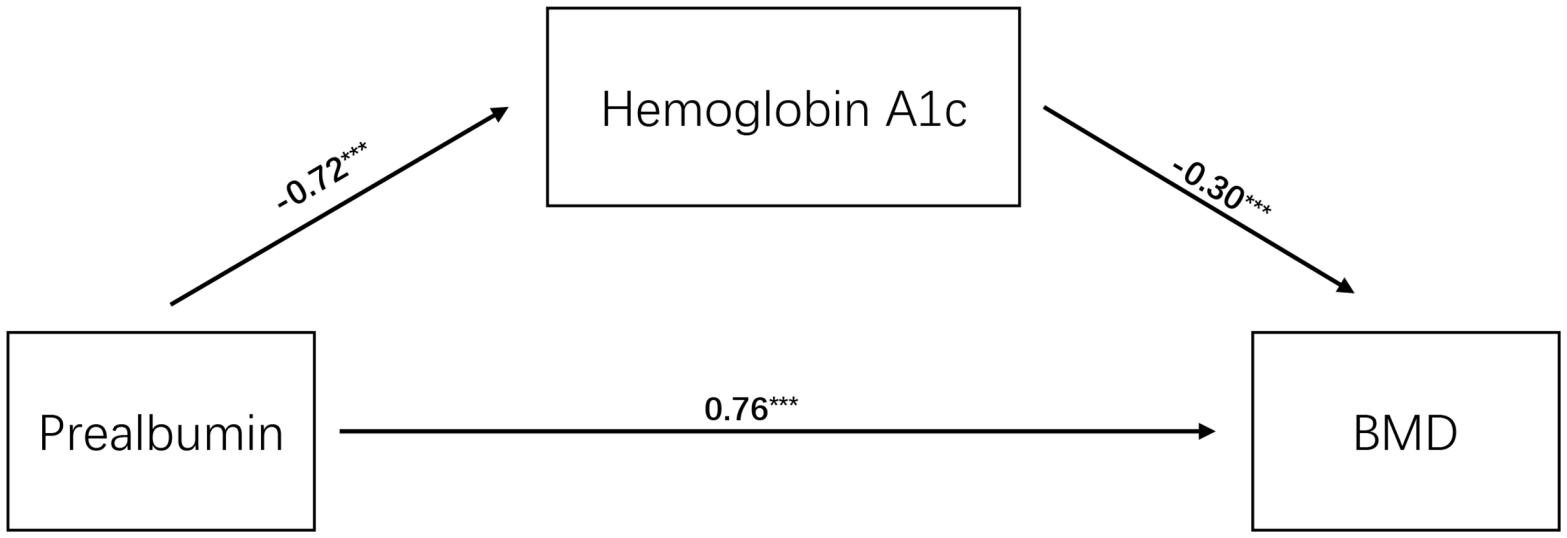
A schematic diagram of the mediating pathway. This analysis indicates a significant indirect effect, suggesting that prealbumin not only directly affects bone density but also indirectly influences BMD through its negative correlation with glycated hemoglobin. Hemoglobin A1c is an intermediate variable between prealbumin and BMD. ***p < 0.001, **p < 0.01, *p < 0.05.

**Figure 6:**
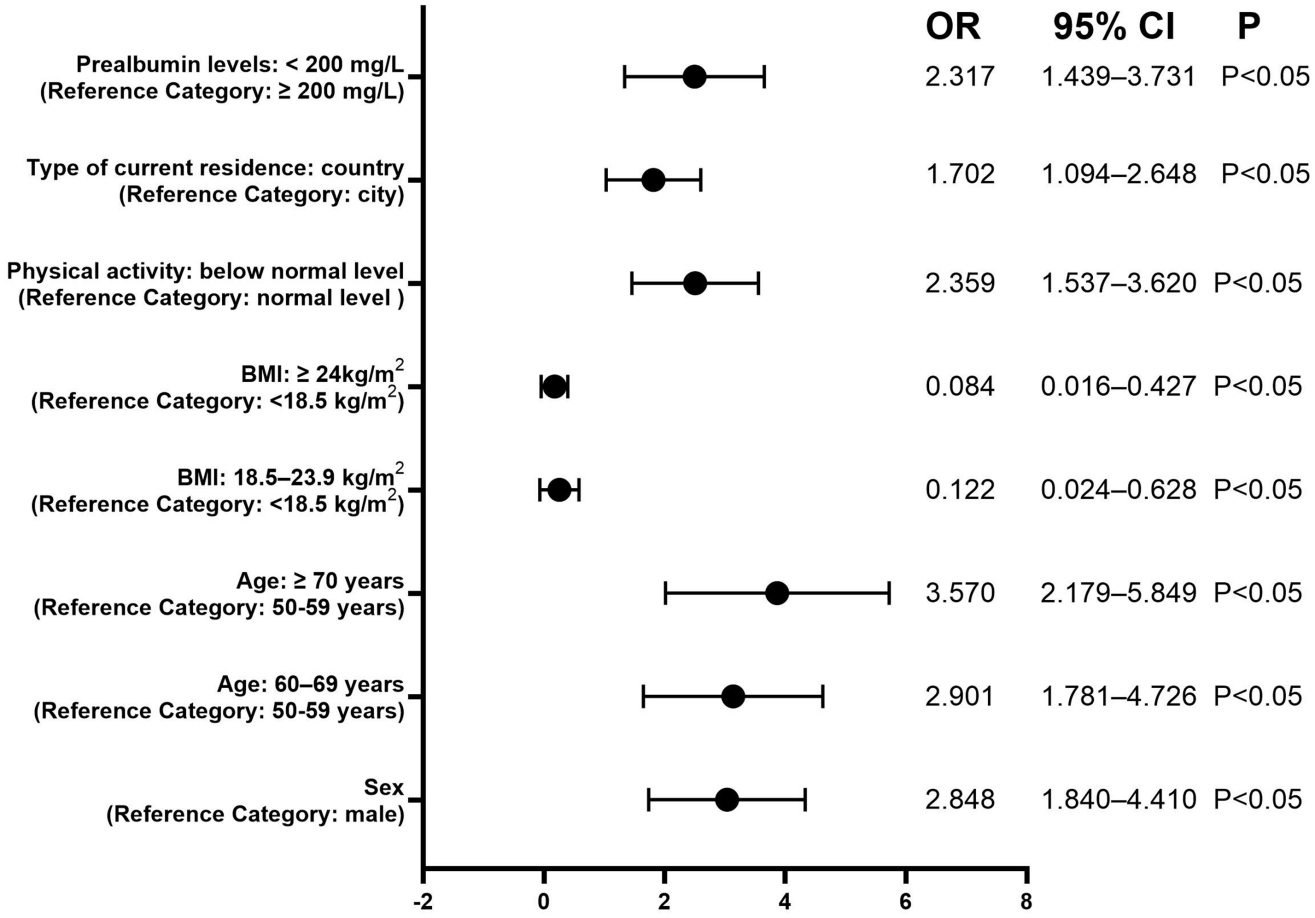
Forest plot of risk factors for osteoporosis. This forest plot displays the odds ratios ORs and 95% CIs derived from a multivariable logistic regression analysis. All variables were mutually adjusted for each other in the model. The reference category for each variable is as indicated.

**Figure 7:**
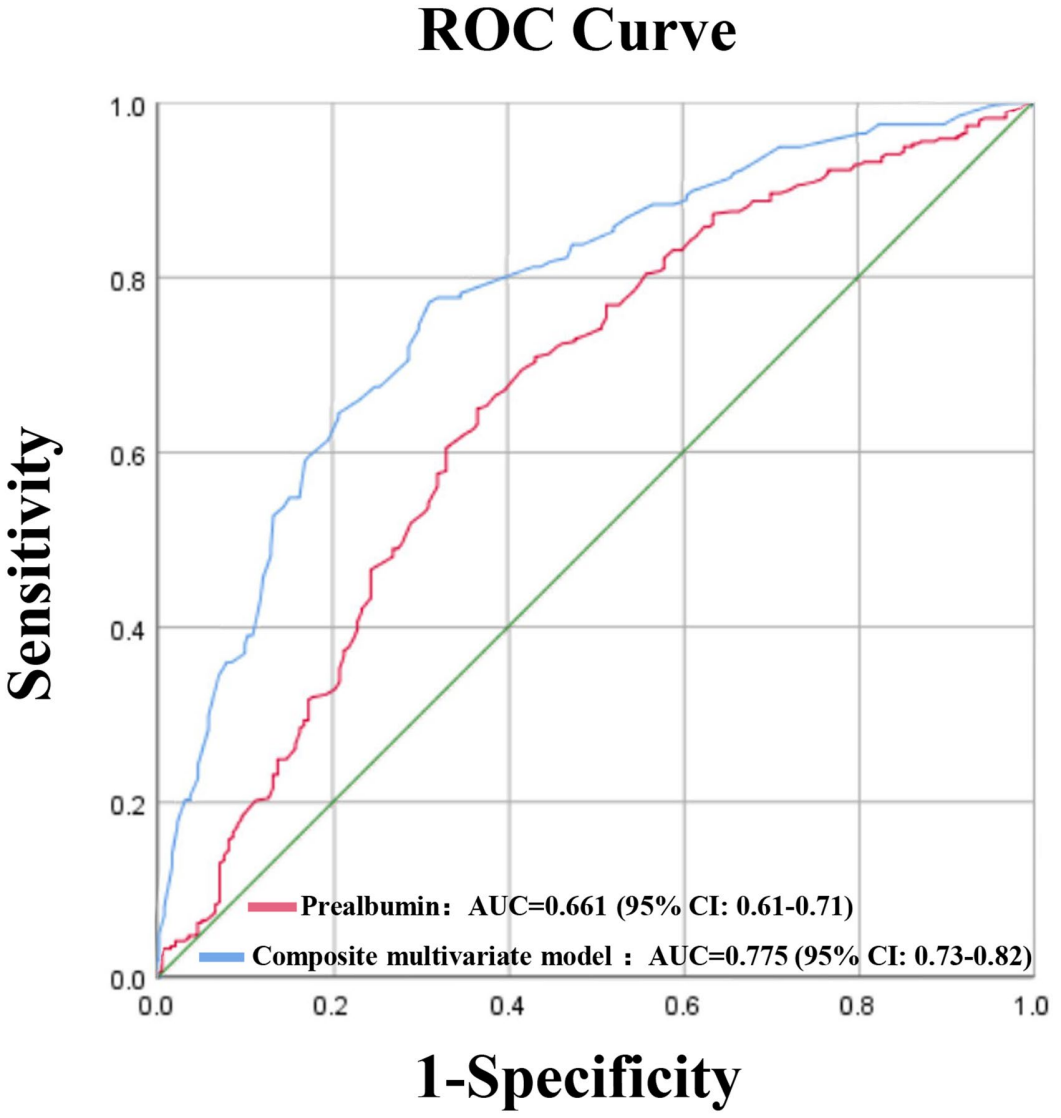
ROC Curves for Predicting Osteoporosis by Prealbumin and a Composite multivariate model. The ROC curves compare the diagnostic efficacy between prealbumin and the composite multivariate model. The composite multivariate model demonstrated superior predictive performance with an AUC of 0.775 (95% CI: 0.73–0.82), significantly higher than prealbumin alone (AUC = 0.661, 95% CI: 0.61–0.71).

A logistic regression model was employed to examine the association between prealbumin levels and osteoporosis risk in adults aged ≥50 years. Specifically, a multivariate model adjusted for key confounders, including age, sex, BMI, living environment, physical activity level, albumin levels, and hemoglobin levels. As a formal mediation analysis confirmed that HbA1c is a significant mediator in the relationship between low prealbumin and osteoporosis, it was justified to exclude HbA1c from the model to avoid over-adjustment bias (Table 5; Figure 5). Results indicated a significant association between low prealbumin levels and increased osteoporosis risk (OR, odds ratio = 2.317; 95% CI, confidence interval: 1.439–3.731; Table 6; Figure 6). Additionally, female sex, lower BMI, advanced age, physical inactivity, hypoalbuminemia, and rural residence were identified as significant risk factors for osteoporosis. Notably, the ROC curve analysis for prealbumin alone yielded an AUC of 0.661 (95% CI: 0.61–0.71), indicating modest discriminatory power as an independent test. The optimal cutoff value was identified at 234.5 mg/L, with a sensitivity of 65% and specificity of 63.5%. In contrast, the ROC analysis for the predicted probability from the composite multivariate model (including age, gender, body mass index, physical activity status, and type of residence) demonstrated a significantly higher AUC of 0.775 (95% CI: 0.73–0.82). The performance of both models is illustrated in Figure 7.

**Table 5:** Mediating role of glycated hemoglobin in the relationship between prealbumin and osteoporosis.

Type of effect	Effect	se	LLCI	ULCI	The proportion mediated (%)
Total effect	0.98	0.17	0.65	1.31	
Direct effect	0.76	0.18	0.42	1.11	77.55
Indirect effect	0.22	0.88	0.07	0.41	22.45

**Table 6:** Odds ratios (95% confidence interval) for osteoporosis according to associated risk factors.

Characteristics	OR	95%CI	p
Sex			
Male	1	(Referent)	<0.05
Female	2.848	1.840–4.410	
Age			
50–59 years	1	(Referent)	<0.05
60–69 years	2.901	1.781–4.726	
≥70 years	3.570	2.179–5.849	
BMI			
Underweight (<18.5 kg/m^2^)	1	(Referent)	<0.05
Normal weight (18.5 kg/m2–23.9 kg/m^2^)	0.122	0.024–0.628	
Overweight (≥24 kg/m^2^)	0.084	0.016–0.427	
Physical activity			
Normal level	1	(Referent)	<0.05
Below normal level	2.359	1.537–3.620	
Type of current residence			
City	1	(Referent)	<0.05
Country	1.702	1.094–2.648	
Prealbumin levels			
Normal level (≥200 mg/L)	1	(Referent)	<0.05
Below normal level (<200 mg/L)	2.317	1.439–3.731	

**Figure 8:**
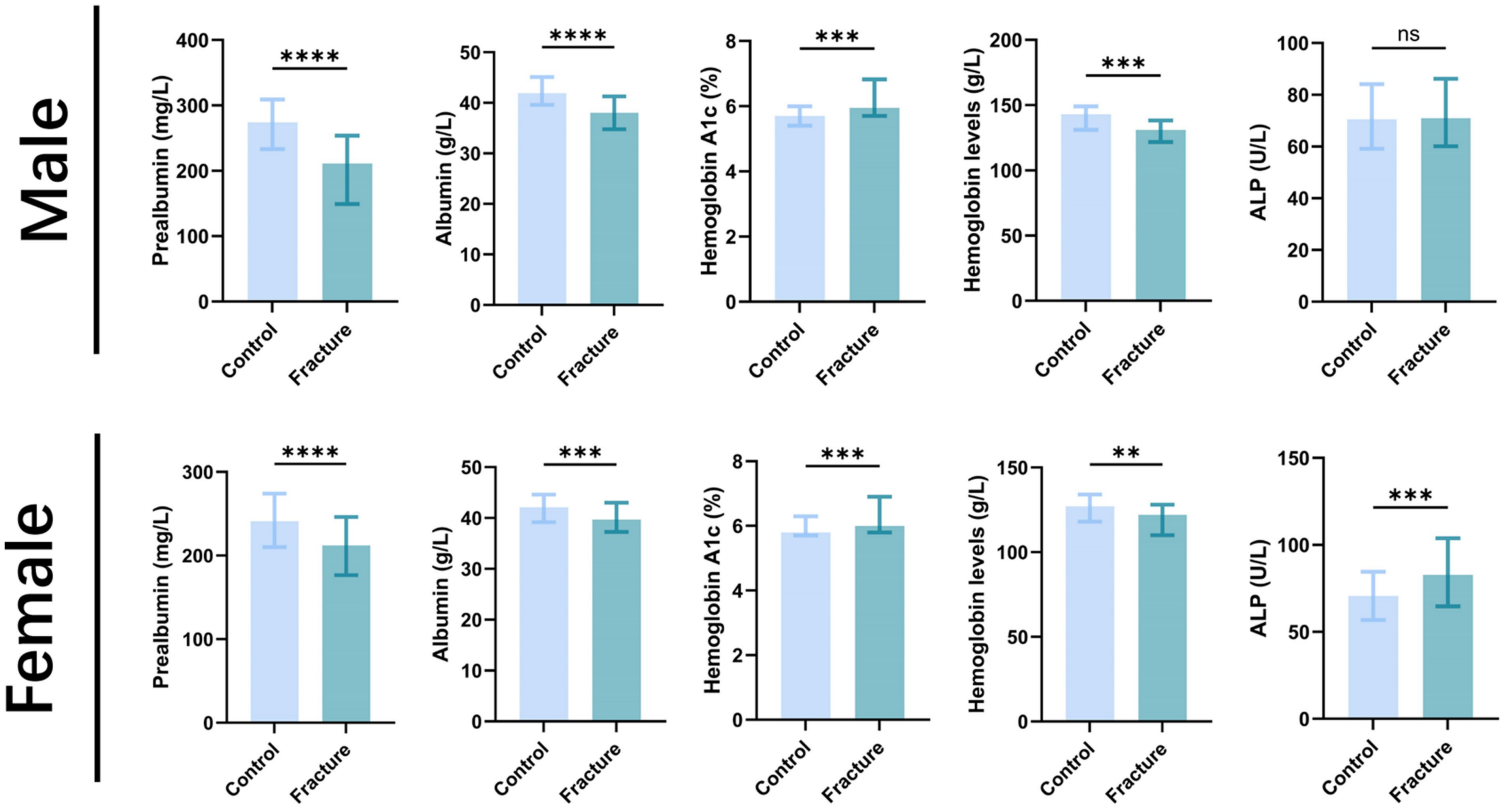
Comparison of biomarker levels between control and fracture groups, stratified by sex. This figure shows the distribution of prealbumin, albumin, hemoglobin A1c, and hemoglobin in the control versus osteoporotic fracture groups, presented separately for males and females. ****p < 0.0001, ***p < 0.001, **p < 0.01, *p < 0.05.

Considering fragility fractures are widely recognized as one of the most severe complications associated with osteoporosis. Therefore, it might have been more clinically relevant to discuss the correlation between low prealbumin levels and the incidence of osteoporotic fractures. To investigate this issue, the participants’ medical records were reviewed again to investigate the occurrence of brittle fractures over a period of nearly 10 years, with a division into control and fracture groups, followed by differential analysis of relevant biochemical indicators. It is worth noting that the findings (Table 7; Figure 8) are consistent with those of previous analyses, indicating that prealbumin not only serves as a significant risk factor for osteoporosis but also holds a predictive value for osteoporotic fractures.

**Table 7:** Biochemical markers of the participants with and without osteoporotic fractures.

Male				
Characteristics	Control group (*n* = 181)	Fracture group (*n* = 26)	*z*	*p*
Prealbumin (mg/L)	274.00 (233.00, 309.00)	211.00 (149.25, 253.75)	−4.380	<0.05
Albumin (g/L)	41.90 (39.60, 45.10)	38.00 (34.75, 41.28)	−4.134	<0.05
Hemoglobin A1c (%)	5.70 (5.40, 6.00)	5.95 (5.7, 5.83)	3.311	<0.05
Hemoglobin levels (g/L)	143.00 (131.00, 149.00)	131.00 (121.75, 138.25)	−3.520	<0.05
ALP(U/L)	70.50 (59.10, 84.15)	70.95 (60.10, 86.18)	0.378	0.705

Female				
Characteristics	Control group (*n* = 251)	Fracture group (*n* = 76)	*z*	*p*
Prealbumin (mg/L)	236.00 (209.00, 269.00)	200.00 (163.25, 233.75)	−5.147	<0.05
Albumin (g/L)	42.10 (39.20, 44.60)	39.70 (37.25, 43.03)	−3.768	<0.05
Hemoglobin A1c (%)	5.80 (5.70, 6.30)	6.00 (5.80, 6.90)	3.441	<0.05
Hemoglobin levels (g/L)	127.00 (118.00, 134.00)	122.00 (110.00, 128.00)	−3.106	<0.05
ALP(U/L)	70.70 (56.80, 84.50)	82.75 (64.63, 103.78)	3.655	<0.05

*Subgroup analysis was conducted based on sex due to the influence of sex on reference values for part markers. Prealbumin, albumin, hemoglobin A1c, and hemoglobin levels, which had a strong association with osteoporotic fractures, remained unaffected by sex.

## 4 Discussion

The present study demonstrated a significant positive correlation between prealbumin levels and BMD, highlighting the crucial role of low prealbumin levels as a determinant of reduced BMD in older adults. In our study, a total of 534 participants were enrolled, including 207 men (prevalence of 23.67%) and 327 women (prevalence of 45.26%), with an overall prevalence of 36.89%. The prevalence rate in this study was consistent with previously reported figures of 22% in men and 40% in women in mainland China (1). It is noteworthy that the significant decline in estrogen levels after menopause in women, a hormone vital for bone protection by inhibiting osteoclast activity and promoting osteoblast function, leads to a key pathophysiological factor: the sharp reduction in estrogen disrupts bone metabolic balance, causing bone resorption to exceed bone formation. This is one of the primary mechanisms driving accelerated bone mineral density loss in women and accounts for the significantly higher prevalence of osteoporosis among female patients compared to males (19).

A recent cross-sectional study found an inverse association between serum 25(OH)D levels and fasting blood glucose levels in patients with osteoporosis, and appropriate vitamin D supplementation lowered blood glucose levels (20). Vitamin D can increase the cytosolic Ca^2+^ level, activating islet *β* exocytosis and increasing insulin secretion (21). In contrast, vitamin D enhances peripheral insulin sensitivity by binding to vitamin D receptors expressed in skeletal muscle and adipose tissue cells (22). Additionally, glucose metabolism disorders affect osteogenesis. High glucose levels selectively enhance autologous Wnt11 expression in mesenchymal stem cells to stimulate adipogenesis and elevate Angiopoietin2 (Ang2) expression through the Wnt/PKC noncanonical pathway. Simultaneously, the Angiopoietin1 (Ang1)-Tyrosine-protein kinase TEK2 (Tie2) signaling pathway is implicated in the proliferation of hematopoietic stem cells in the bone marrow. Excessive Ang2 expression disrupts this mechanism, consequently reducing the stem cell population (given that Ang1 and Ang2 bind to the same Tie2 receptor, they antagonize each other). This results in impaired osteogenesis and bone loss (23). Simultaneously, abnormal adipocyte generation influences the atherogenic index of plasma via multiple mechanisms, including lipid metabolic disturbances, inflammatory responses, and insulin resistance. It is important to note that the atherogenic index of plasma levels associated with decreased trabecular bone score (24, 25). In a separate study conducted by our research group, we also observed that a drug utilized for the treatment of atherosclerosis can effectively suppress osteoclast differentiation. Nevertheless, the precise molecular mechanisms underlying this effect warrant further detailed investigation. Additionally, chemical cross-linking can also occur between serum sugars and exposed amino acid residues, forming advanced glycation end products (26). Advanced glycation end products elicit an inflammatory response and contribute to bone resorption, thereby increasing the susceptibility to osteoporosis (27). Therefore, hyperglycemia is an important risk factor for osteoporosis.

**Figure 9:**
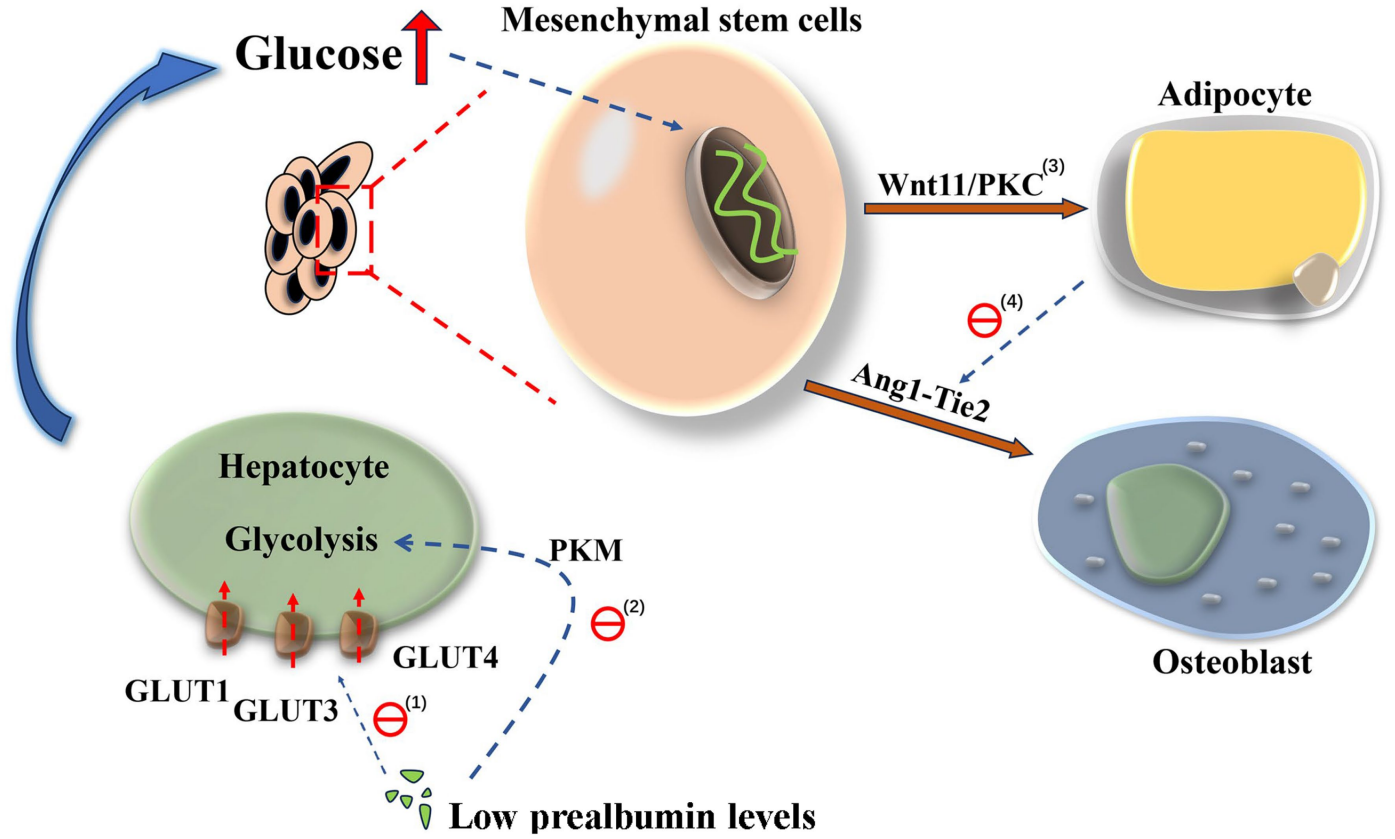
Low levels of prealbumin mediate the differentiation of mesenchymal stem cells into adipocytes by increasing blood glucose. Original. (1) Low prealbumin levels inhibits the uptake of glucose via GLUT1, GLUT3, and GLUT4. (2) Low prealbumin levels interferes with glycolysis by decreasing the expression of pyruvate kinase M. (3) High glucose activates the Wnt11/PKC pathway and induces the differentiation of mesenchymal stem cells to adipocytes. (4) Adipocytes possess the capacity to upregulate Ang2 production, leading to an imbalance in Ang1: Ang2 levels, inhibition of the Ang1-Tie2 signaling pathway, and disruption of mesenchymal stem cells differentiation into osteoblasts. Abbreviations: GLUT1, glucose transporter type 1; GLUT3, glucose transporter type 3; GLUT4, glucose transporter type 4; Ang1, Angiopoietin1; Ang2, Angiopoietin2.

An inverse correlation was observed between prealbumin and hemoglobin A1c levels in our study participants. As mentioned in past studies, prealbumin not only serves as a crucial component in the normal coupling of stimulus-secretion in pancreatic *β*-cells but also plays a vital role in maintaining the integrity of β-cells. It lowers blood glucose levels by directly acting on glucose-induced electrical activity and voltage-gated Ca^2+^, promoting a glucose-induced increase in free cytosolic Ca^2+^ concentration and insulin release (28). Moreover, the expression of glucose transporters has been found to be influenced by prealbumin levels, and a deficiency in prealbumin can inhibit the expression of glucose transporter type 1 (GLUT1), glucose transporter type 3 (GLUT3), and glucose transporter type 4 (GLUT4), thereby reduce cellular glucose uptake and increase blood glucose levels. Insufficient prealbumin levels can concurrently result in the downregulation of pyruvate kinase M expression and inhibition of glycolysis (29). Therefore, we postulated that prealbumin might induce hepatic metabolic dysfunction characterized by elevated extracellular or plasma glucose levels and reduced glucose transporter influx (see Figure 9 for a possible interpretation of our results).

Serum albumin and prealbumin play a crucial role in the nutritional assessment of the body. Low levels of albumin are often associated with chronic inflammation, cancer, liver disease, kidney disease syndrome, and malnutrition, which are all systemic diseases. Furthermore, studies have also indicated a correlation between low levels of serum albumin and osteoporosis (OR: 4.59, 95% CI: 1.49–14.16, *p* = 0.025) (15). We observed similar results showing that low levels of albumin are more likely to contribute to the development of osteoporosis, although the underlying mechanism remains unclear.

Considering the liver as the primary source of prealbumin, we investigated the impact of hepatic enzymes on osteoporosis occurrence. ALP was selected for the analysis, yielding results consistent with previous research findings. Specifically, serum ALP was found to have a negative correlation with BMD (30). The Mendelian randomization analysis further demonstrated a robust causal association between ALP and BMD (31). Moreover, this discrepancy was exclusively observed among female participants in our study, which may be attributed to abnormally elevated ALP levels resulting from accelerated bone metabolism in postmenopausal women. Notably, an abnormal elevation in ALP levels will heighten the risk of bone fractures (OR: 1.49, 95% CI: 1.07–2.07) (32). The validity of this association was further confirmed in subsequent analyses.

Meanwhile, we believe that the development of osteoporosis is significantly associated with inflammation and that the decrease in prealbumin levels is also closely related to inflammation. Inflammatory factors not only reduce prealbumin synthesis in the liver but also cause hemodilution and dilation of the extravascular space, leading to a decrease in prealbumin levels. A recent study on older male patients without renal or hepatic impairment confirmed that low prealbumin and albumin levels are mainly caused by inflammation (33). In contrast, in both chronic and acute stress conditions, the synthesis of plasma prealbumin is downregulated, whereas body nitrogen stores are depleted owing to the combined effects of cytokines on hepatic and muscular tissues. The reduction in plasma prealbumin levels observed in most inflammatory disorders indicates the depletion of endogenous nitrogen stores through the urinary excretion of nitrogenous compounds (34). Of note is the inflammatory factor interleukin-17 (IL-17); which induces innate immune cells to secrete more receptor activator of nuclear factor-κB ligand (RANKL), tumor necrosis factor alpha, and interleukin-1, thereby promoting bone resorption. Osteocytes secrete RANKL ligands that bind to its receptor, receptor activator of nuclear factor-κB, and regulate osteoclast proliferation, differentiation, and activity (35). Additionally, the mechanism of action of IL-17 in reducing prealbumin levels remains to be studied further; however, a significant reduction in prealbumin levels in patients with high IL-17 expression was mentioned in a previous study. Researchers believe that IL-17 is significantly associated with disease progression, systemic inflammation, cellular immune suppression, and malnutrition (36). Therefore, we believe that a low prealbumin level is a critical marker for osteoporosis.

Our ROC curve analysis yielded a key clinical insight regarding the role of prealbumin. As a standalone biomarker, prealbumin demonstrated limited discriminatory power (AUC = 0.66), confirming its inadequacy for definitive diagnosis of osteoporosis—a finding consistent with the disease’s complex pathogenesis. However, when integrated into a composite model, its utility was markedly enhanced, as reflected in a significantly superior AUC of 0.775. This indicates that the primary value of prealbumin lies not in its use as a solitary test, but as an efficient and cost-effective component within a multifactorial risk assessment tool. The implementation of such a composite score in primary care could be instrumental for risk stratification and for guiding referrals for definitive DXA testing, thereby optimizing resource allocation.

Finally, it is important to acknowledge the limitations of this study. As mentioned earlier, fragility fractures are widely recognized as one of the most severe complications associated with osteoporosis. Utilizing the fracture risk assessment tool (FRAX score) to stratify participants and investigating the association between low prealbumin levels and the incidence of osteoporotic fractures would significantly enhance the clinical relevance of this study. The FRAX score is a more comprehensive and reliable fracture risk prediction tool, which can provide timely guidance for preventing fractures in patients. As this was a retrospective study, the use of FRAX score grouping may introduce potential bias caused by subjective factors from participants, and we also encountered missing data related to the FRAX score. Taking all the above factors into account, we did not incorporate the FRAX score into our analysis. However, by conducting osteoporosis risk factor screening from the perspective of biochemical indicators in the population, we not only effectively addressed the limitation of the FRAX score in biochemical assessment but also provided a brand-new perspective for a deeper understanding of the pathogenesis of osteoporosis. Furthermore, our study relied solely on BMD measurements from the lumbar spine. Future studies should incorporate BMD data from multiple skeletal sites (particularly the femoral neck) to verify whether the impact of low prealbumin levels on bone health is systemic or site-specific. In order to further explore the association between prealbumin levels and osteoporosis, our upcoming prospective cohort study will systematically recruit middle-aged and elderly participants and stratify them based on their prealbumin levels. We will conduct baseline assessments and follow up for 3 to 5 years to monitor changes in bone density and the incidence of fractures. And we will further investigate the molecular aspects of bone metabolism to elucidate the mechanism by which low prealbumin levels cause osteoporosis, so as to prevent and inform the relevant diagnosis and treatment of osteoporosis.

## 5 Conclusion

Although the relationship between prealbumin and osteoporosis remains controversial, several studies have shown that prealbumin levels can be used to effectively predict the occurrence of osteoporosis and that the significant association between prealbumin and osteoporosis should not be ignored. Our study showed that prealbumin is not only a marker of nutritional status but also has a correlation with blood glucose levels, lipid metabolic disorders induced by hyperglycemia, and inflammatory factor activity in the body. A low prealbumin level may predispose individuals to osteoporosis.

## Data Availability

The original contributions presented in the study are included in the article/supplementary material, further inquiries can be directed to the corresponding author/s.
